# Death of Prof. Richard O. Cowling, A. M., M. D.

**Published:** 1881-04

**Authors:** 


Death of Prof. Richard O. Cowling, A. M., M. D.—We were pained to learn, just as our editorial department was being closed for the present issue, of the sad and untimely demise of the distinguished gentleman and enlightened physician whose name appears at the caption of this paragaph.

Though personally unknown to us, we learned to admire, and then to love him for his bold and uncompromising defense of the rights and the dignity of the medical profession; a profession to which he voluntarily and willingly consecrated his great and cultured intellect. But he is gone to his reward above. He died as he had lived for a long while, in full and implicit faith in the world's Redeemer. We make some extracts from the *Louisville Medical News*, which was established by him, and which he nobly edited until death called him from his labors, to which the attention of our readers is invited. He was a native of South Carolina, having been born near Georgetown in that State on the 9th of April, 1839. He was thoroughly educated in and out of his profession, having graduated from Trinity College, Connecticut, and Jefferson Medical College, Philadelphia.

